# Abnormal Topological Organization of Sulcal Depth-Based Structural Covariance Networks in Parkinson's Disease

**DOI:** 10.3389/fnagi.2020.575672

**Published:** 2021-01-15

**Authors:** Erlei Wang, Yujing Jia, Yang Ya, Jin Xu, Chengjie Mao, Weifeng Luo, Guohua Fan, Zhen Jiang

**Affiliations:** ^1^Department of Radiology, The Second Affiliated Hospital of Soochow University, Suzhou, China; ^2^Department of Neurology, The Second Affiliated Hospital of Soochow University, Suzhou, China

**Keywords:** magnetic resonanc imaging, graph theoretical analysis, sulcal depth, structural covariance networks, Parkinson's disease

## Abstract

Recent research on Parkinson's disease (PD) has demonstrated the topological abnormalities of structural covariance networks (SCNs) using various morphometric features from structural magnetic resonance images (sMRI). However, the sulcal depth (SD)-based SCNs have not been investigated. In this study, we used SD to investigate the topological alterations of SCNs in 60 PD patients and 56 age- and gender-matched healthy controls (HC). SCNs were constructed by thresholding SD correlation matrices of 68 regions and analyzed using graph theoretical approaches. Compared with HC, PD patients showed increased normalized clustering coefficient and normalized path length, as well as a reorganization of degree-based and betweenness-based hubs (i.e., less frontal hubs). Moreover, the degree distribution analysis showed more high-degree nodes in PD patients. In addition, we also found the increased assortativity and reduced robustness under a random attack in PD patients compared to HC. Taken together, these findings indicated an abnormal topological organization of SD-based SCNs in PD patients, which may contribute in understanding the pathophysiology of PD at the network level.

## Introduction

Parkinson's disease is a chronic neurodegenerative disease, characterized by a diverse array of motor and non-motor symptoms (Sveinbjornsdottir, 2016). The main pathological feature of PD is the progressive apoptosis of dopaminergic neurons in the substantia nigra pars compacta due to the abnormal intracellular accumulation of α-synuclein known as Lewy pathology. As the disease progresses, Lewy pathology gradually spreads from mid-brain nucleus to widespread cortical regions leading to structural abnormalities (Braak et al., 2003; McCann et al., 2016). Currently, many sMRI studies have found significant abnormalities in a wide range of brain regions in PD patients using different morphometric features such as gray matter volume (Camicioli et al., 2009; Pan et al., 2012), cortical thickness (Ibarretxe-Bilbao et al., 2012; Uribe et al., 2018), local gyrification index (LGI) (Zhang et al., 2014; Sterling et al., 2016), and local fractional dimension (Li et al., 2020). However, these studies can only reflect the localized changes, and the interrelationship of the altered brain regions remains largely unknown.

The brain regions are highly interconnected by axonal connectivity and would covary in the morphological features as a result of sharing trophic, genetic, and neurodevelopmental influences (Alexander-Bloch et al., 2013). Based on this key foundation, graph theoretical analysis has been successfully used to study the topological abnormalities of structural covariance networks (SCNs) in various neurological conditions, such as schizophrenia (Zhang et al., 2012; Palaniyappan et al., 2015, 2019), Alzheimer's disease (He et al., 2008; Friston et al., 2010; Pereira et al., 2016), and amyotrophic lateral sclerosis (Zhang et al., 2019). This methodology provides a powerful tool for investigating the neurobiological network mechanisms of different diseases by using a series of quantitative parameters (e.g., small-world property, modularity, hub analysis, degree distribution, and robustness analysis) (Rubinov and Sporns, 2010; Hosseini et al., 2012). So far, using different morphometric features, some studies have applied this methodology to investigate the alterations of SCNs in PD patients, but the results are inconsistent. For example, some studies have found a significantly increased clustering coefficient and path length in PD patients compared to healthy controls (HC) (Pereira et al., 2015; Zhang et al., 2015; Xu et al., 2017; Wu et al., 2018), while others found no changes at all (Guo et al., 2018; Xu et al., 2018). Brain network analyses using diffusion tensor imaging (DTI) have also reported mixed results with some reporting lower global efficiency (the inverse of path length) and lower clustering coefficient in PD patients compared to HC (Kamagata et al., 2018; Vriend et al., 2018; Koirala et al., 2019; Hu et al., 2020), while others found no differences (Tinaz et al., 2017; Kok et al., 2020).

Sulcal depth (SD) is a gyrification feature defined as the Euclidean distance between the central surface and its convex hull (Yun et al., 2013). Distinct from gray matter volume and cortical thickness, SD can provide the information about the shape of cortical surface. SD gradually decreased with aging (Jin et al., 2018) and has been used as a sensitive and reliable indicator in Alzheimer's disease (Im et al., 2008; Yun et al., 2013), schizophrenia (Lyu et al., 2018; Yan et al., 2019), Williams Syndrome (Kippenhan et al., 2005), and anorexia nervosa (Nickel et al., 2019). It has been shown that shallower SD was associated with the combined effects of decreased cortical thickness and gyral white matter volume (Im et al., 2008). On the other hand, abnormal SD changes may also result from altered corticortical connections according to the tension-based theory of cortical folding (Van Essen, 1997). Hence, we speculate that SD may be a suitable and sensitive morphometric feature for SCNs studies, especially in PD patients with obvious gray and white matter pathology (Braak et al., 2003; McCann et al., 2016). To the best of our knowledge, the SD-based SCNs have not been investigated in PD patients. In addition, although there have been some studies investigating the topological properties of SCNs in PD patients, little is known about the degree distribution and assortativity of PD networks.

Therefore, in the present study, we used SD and graph theoretical analysis to explore topological abnormalities of SCNs in 60 PD patients compared with 56 age- and gender-matched HC. A series of global network parameters, regional network parameters, hub analysis, degree distribution and network robustness were computed and compared between the two groups. Based on previous findings, we hypothesized that PD patients would show increased clustering coefficient and path length, as well as a reorganization of regional network parameters and hubs.

## Materials and Methods

### Participants

The present study was approved by the Medical Research Ethical Committee of The Second Affiliated Hospital of Soochow University. Written informed consent was obtained from all participants before evaluation. All participants were right-handed. A total of 60 PD patients were recruited by an experienced neurologist according to the UK Brain Bank criteria (Hughes et al., 1992). The patient exclusion criteria included atypical parkinsonian disease, severe neurological or psychiatric comorbidity, history of alcohol or substance abuse, Mini-Mental State Examination (MMSE) score ≤25 and any MRI contraindications. The Unified Parkinson's Disease Rating Scale motor section (UPDRS-III), Hoehn and Yahr (H&Y) staging scale and MMSE score were used to assess the motor symptom, the severity of the disease and the global cognitive function, respectively. All PD patients were assessed and MRI scanned in the ON medication state. Levodopa equivalent daily dose for each patient was calculated following established guidelines (Tomlinson et al., 2010).

An age- and gender-matched group of 56 healthy controls (HC) with no history of neurological or psychiatric diseases was enrolled in the study. None of the HC showed gross abnormalities in structural MRI. The demographic and clinical indices of all participants are shown in Table 1.

**Table 1:** Demographic and clinical details for each group.

	**HC (*n* = 56)**	**PD (*n* = 60)**	***P***
Age (years)	63.07 ± 5.543	61.60 ± 6.9	0.211
Gender(male/female)	(31/25)	(30/30)	0.564
Education (years)	8.55 ± 3.94	7.88 ± 3.76	0.351
Duration of illness (years)		3.73 ± 2.05	
UPDRS III score		22.55 ± 12.52	
HandY		1.91 ± 0.63	
MMSE score	28.36 ± 0.98	28.17 ± 1.21	0.355
LEED (mg)		394.37 ± 137.25	

*PD, Parkinson's disease; HC, healthy control; UPDRS-III, Unified Parkinson's Disease Rating Scale (motor section); H&Y, Hoehn and Yahr staging; MMSE, Mini-Mental State Examination; LEED, levodopa equivalent daily dose*.

### MRI Data Acquisition

Anatomical 3D T1-weighted fast field echo (FFE) MRI images were acquired on a 3T Philips Achieva scanner (Philips, Best, The Netherlands) using a 32-channel receive coil in the Department of Medical Imaging, The Second Affiliated Hospital of Soochow University. A memory foam padding was used to minimize head motion, and earplugs were used to reduce scanner noise. The MRI parameters were as follows: 155 sagittal slices, repetition time (TR) = 7.1 ms, echo time (TE) = 3.5 ms, thickness = 1.0 mm, no gap, flip angle 8°, matrix size 220 × 199 reconstructed to 352 × 352 over a 220-mm field of view, and voxel size = 0.625 × 0.625 × 1 mm^3^.

### Data Processing

The MRI data preprocessing was conducted using the Computational Anatomy Toolbox 12 (CAT12, http://dbm.neuro.uni-jena.de/cat12/, version r1434) in a standard manner. All images saved as DICOMs were converted to Nifti-format, and then were inspected visually for motion or other artifacts using the MRIcron software (http://people.cas.sc.edu/rorden/mricron/index.html). Image preprocessing included correction for bias-field inhomogeneities; tissue segmentation into gray matter, white matter, and cerebrospinal fluid; and normalization using DARTEL algorithm. After the preprocessing was finished, only participants receiving a weighted average score of B+ or higher were included for further analysis by viewing the quality reports obtained by CAT12.

The SD was computed according to the manual established by Gaser and Kurth (http://dbm.neuro.uni-jena.de/cat12/CAT12-Manual.pdf). Firstly, using projection-based thickness (PBT) method (Dahnke et al., 2013), the central surface of cortex was reconstructed, which was used as input for calculating SD. Then, we respectively extracted the SD of both hemispheres using the “Extract Additional Surface Parameters” provided in CAT12. SD was estimated based on the Euclidean distance between the central surface and its convex hull (Yun et al., 2013). Finally, according to the Desikan–Killiany (DK40) atlas, 68 parcellated brain regions were extracted as the average SD of all vertices belonging to the region with the “ROI Tools” implemented in CAT12 (Table 2).

**Table 2:** Cortical regions of the Desikan–Killiany atlas.

**Region**	**Abbreviations**	**Index (left)**	**Index (right)**
Banks of the superior temporal sulcus	bSTS	1	35
Caudal anterior cingulate	CAR	2	36
Caudal middle frontal gyrus	cMFG	3	37
Cuneus	CUN	4	38
Entorhinal cortex	EC	5	39
Fusiform gyrus	FG	6	40
Inferior parietal gyrus	IPG	7	41
Inferior temporal gyrus	ITG	8	42
Isthmus cingulate	IC	9	43
Lateral occipital gyrus	LOG	10	44
Lateral orbitofrontal gyrus	LFGor	11	45
Lingual gyrus	LG	12	46
Medial orbitofrontal gyrus	MFGor	13	47
Middle temporal gyrus	MTG	14	48
Parahippocampal gyrus	ParaHIPP	15	49
Paracentral gyrus	ParaCG	16	50
Pars opercularis	pOPER	17	51
Pars orbitalis	pORB	18	52
Pars triangularis	pTRI	19	53
Pericalcarine cortex	PeriCAL	20	54
Postcentral gyrus	PostCG	21	55
Posterior cingulate	PCC	22	56
Precentral gyrus	PreCG	23	57
Precuneus	PreCUN	24	58
Rostral anterior cingulate	RAC	25	59
Rostral middle frontal gyrus	rMFG	26	60
Superior frontal gyrus	SFG	27	61
Superior parietal gyrus	SPG	28	62
Superior temporal gyrus	STG	29	63
Supramarginal gyrus	SupraMG	30	64
Frontal pole	Fpole	31	65
Temporal pole	Tpole	32	66
Transverse temporal gyrus	TTG	33	67
Insula	INS	34	68

### Constructing Structural Covariance Networks

**Figure 1:**
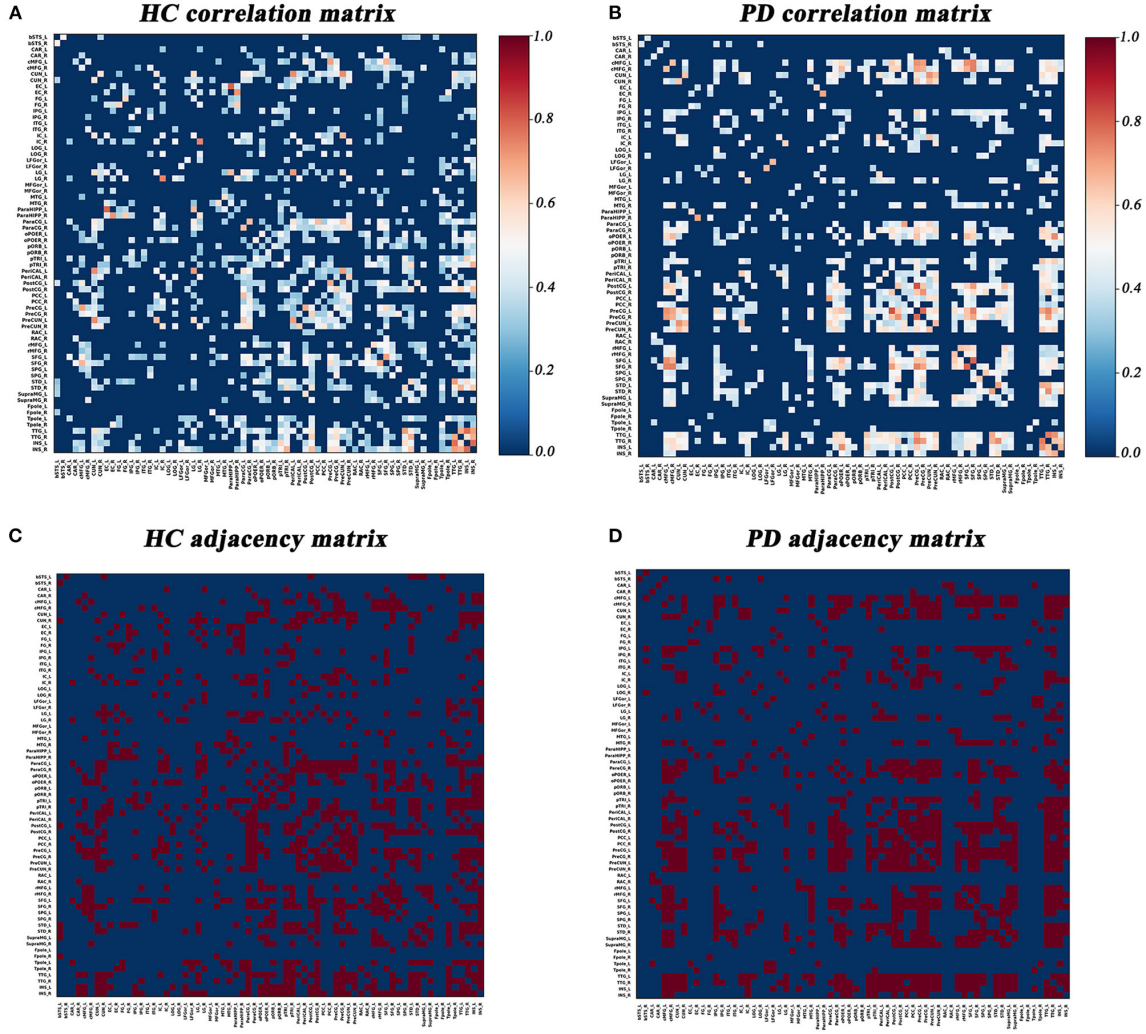
Correlation matrices and adjacency matrices with 68×68 for healthy controls (HC) and PD patients. Correlation matrices for HC **(A)** and PD patients **(B)**, and binary adjacency matrices at the minimum density (0.26) for HC **(C)**, and PD patients **(D)**. Correlation matrices show the Pearson correlation coefficient between any two regions of the network and the color bar denotes the absolute value of the Pearson correlation coefficient and represents the strength of the connections.

Graph Analysis Toolbox (GAT) was used to construct the SD-based SCNs (Hosseini et al., 2012). For each group, a 68×68 correlation matrix was established by calculating Pearson correlation coefficients between SD values of each brain region adjusted for age and gender. Thereafter, the correlation matrix was converted into a binary adjacency matrix by thresholding correlation coefficients into values of 1 or 0 (Figure 1). Here, these thresholds were defined as a range of network densities varying from 0.26 to 0.5 (increments of 0.02), which ensured that PD and HC networks had the same number of nodes and edges at each density. The minimum density (0.26) was determined to ensure that the networks were not fragmented for both groups. For density above 0.5 the networks approached random configuration (Humphries et al., 2006; Singh et al., 2013).

### Network Parameters

A series of global and regional network parameters was employed to characterize the topological properties of the SD-based SCNs. The definitions of these parameters are the same as previous studies (He et al., 2008; Rubinov and Sporns, 2010; Zhang et al., 2019). Global network parameters include normalized clustering coefficient, normalized path length, and small-world index. Briefly, the clustering coefficient (Cp) of a node pertains the number of existing connections linking the neighbors of the node divided by all their possible connections. The Cp of a network is the average of clustering coefficients across all nodes in a network, which represents network segregation. The shortest path length (Lp) is equal to the minimum number of edges that connect two nodes. The Lp of a network pertains to the average shortest path length involving all node pairs in the network, which represents network integration.

Small-world index is characterized by a high Cp (>1) and a similar Lp (≈1) compared with the random networks, which represents an optimal balance between network segregation and integration (Watts and Strogatz, 1998). The Cp and Lp of real networks (HC and PD) were normalized to the mean values of 20 random networks constructed with the same number of nodes, edges, and degree distribution as the real networks. Small-world index was calculated by the ratio of normalized clustering coefficient to normalized path length (>1) (Humphries et al., 2006).

Regional network parameters include clustering coefficient, degree, and betweenness. Degree, a measure of a node' s interaction within the network, is the number of connections that the node has with all other nodes in the network. Betweenness is the fraction of all shortest paths in the network that run through a given node.

### Hub Analysis

Hubs are the most globally interconnected regions in a network and were defined as a region whose nodal degree or betweenness value was at least one and a half standard deviation larger than the mean value (He et al., 2008).

### Degree Distribution

Degree distribution reveals specific characteristics of the network in terms of possibility of having high-degree regions and robustness to network damage (Albert et al., 2000; He et al., 2007). It has been shown that human SCNs follow an exponentially truncated power-law distribution (He et al., 2007), suggesting a network that is comprised of most nodes with a degree value close to the mean and also some high-degree nodes with many connections. Such degree distribution is formulated as: *P*(d)~[d^(k/1)^^*^ exp(–d/dc)], where *P*(d) is the probability of network regional degree (d), k is the power exponent and dc is the cut-off degree above which there is an exponential decay in probability of high-degree nodes (Hosseini et al., 2016).

### Network Robustness

Assortativity is a parameter to see if nodes with similar degree tend to be interconnected. In general, a network is degree assortative if high-degree nodes are connected to other nodes with high-degree, while it is degree disassortative if high-degree nodes are connected to other nodes with low-degree nodes (Newman, 2002). Assortativity provides information about connections between nodes and robustness of the network.

Resilience of the network was assessed by a random or targeted attack. In a random attack, nodes were deleted randomly from a network and the size of the largest connected component of the resulting networks was calculated (Bernhardt et al., 2011; Hosseini et al., 2016). This simulation was done 1,000 times to obtain the average measures of the remaining network (Bernhardt et al., 2011; Hosseini et al., 2016). As for a targeted attack, the above processes were repeated, but removing nodes from a network in order of their degrees, from highest to lowest (Bernhardt et al., 2011; Hosseini et al., 2016). We also calculated the area under the curve (AUC) to indicate the aggregate metrics of network robustness.

### Statistical Analyses

The statistical analyses of demographic and clinical indices were performed using the SPSS 22.0 (SPSS Inc., Chicago, IL, USA). Demographic and clinical indices were analyzed with Student's *t*-test for continuous variables and the Chi Square test for categorical variables. Statistical significance was set to *P* < 0.05.

To assess the statistical significance of between-group differences in all network parameters, we used a non-parametric permutation test with 1,000 repetitions (He et al., 2008; Zhang et al., 2019). For each repetition, the corrected SD values of each subject were randomly reassigned to one of two new groups with the same number as the original PD and HC groups, and then the correlation matrices were recalculated for the two new groups. Subsequently, binarized matrices were generated using a range of network densities (0.26–0.5, increments of 0.02). For the two new groups, network parameters were calculated and differences were compared at each density. A permutation distribution of difference was generated under the null hypothesis. The actual difference between PD patients and HC was placed in the corresponding permutation distribution and a 1-tailed *P*-value was calculated based on percentile position. An AUC summary measure was used to evaluate the between-group differences across all densities (Zhang et al., 2019). The statistical threshold was set at *P* < 0.05 for group differences in global network parameters. *P* < 0.05 was deemed significant after false discovery rate (FDR) correction for the regional network parameters.

## Results

### Demographic and Clinical Characteristics

No significant differences were found between HC and PD patients in age (*P* = 0.211), gender (*P* = 0.564), education (*P* = 0.351), and MMSE score (*P* = 0.355) (Table 1).

### Alterations in Global Network Parameters

**Figure 2:**
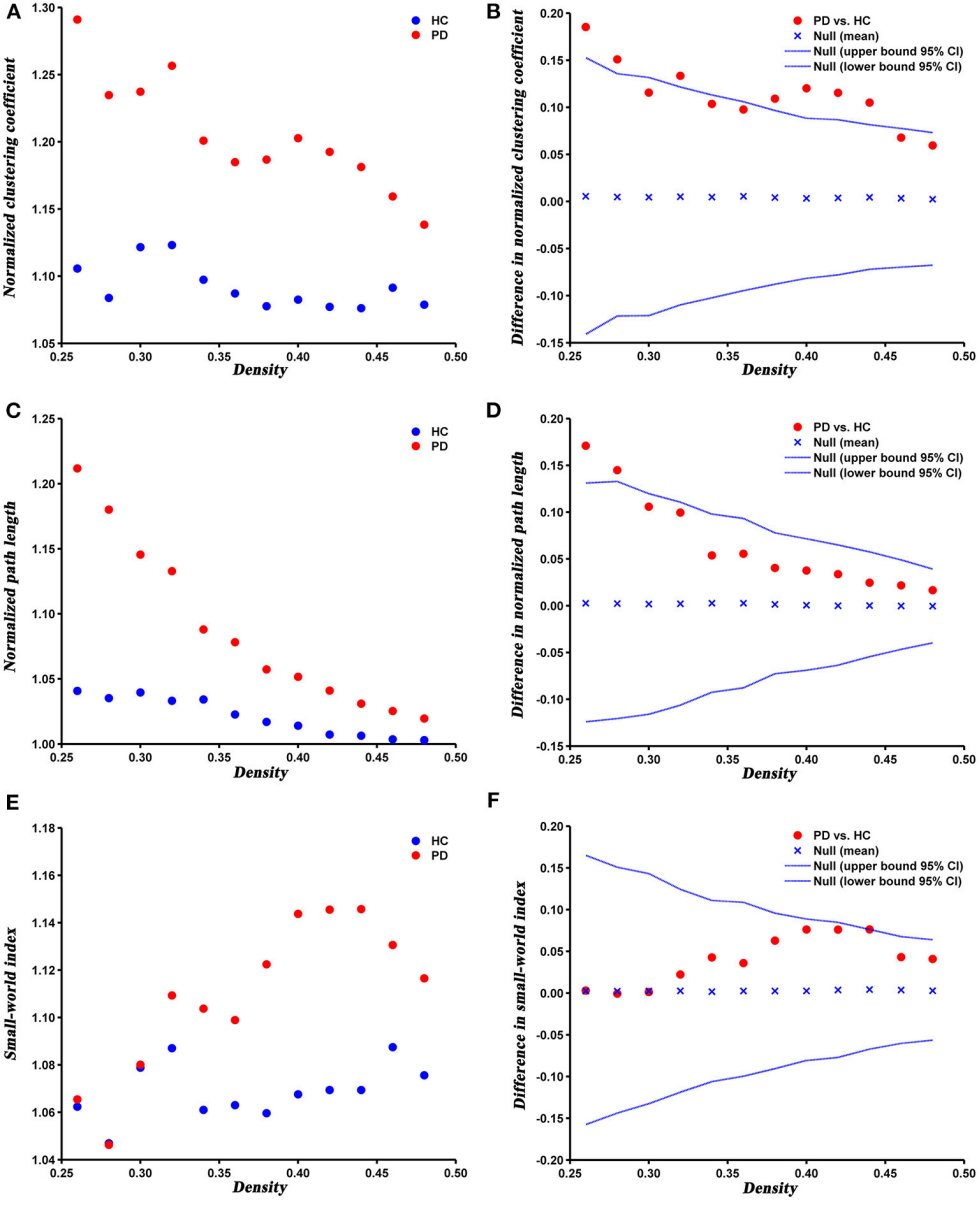
Changes and between-group differences of normalized clustering coefficient **(A,B)**, normalized path length **(C,D)**, and small-world index **(E,F)** as a function of network density in HC and PD groups. Between-group differences (Dot markers) lying outside the 95% confidence intervals (dashed lines) indicate the densities where the difference is significant at *P* < 0.05. Positive values indicate densities where values for PD patients are greater than for HC and negative values indicate the opposite. All abbreviations were listed in Table 2.

The changes and between-group differences in global network parameters of PD and HC groups at densities ranging from 0.26 to 0.50 are shown in Figure 2. We found that the SCNs of both groups showed a small-world property, with normalized clustering coefficient > 1, normalized path length ≈ 1, and the small-world index > 1. Compared with HC, PD patients exhibited increased normalized clustering coefficient, normalized path length, and small-world index at several network densities. The AUC analysis revealed that normalized clustering coefficient and normalized path length were significantly increased (*P* = 0.005 and *P* = 0.042, respectively) in PD patients compared to HC at a range of densities (0.26–0.5).

### Alterations in Regional Network Parameters

The AUC analysis found no significant between-group differences in regional network parameters after FDR correction.

### Network Hubs

**Figure 3:**
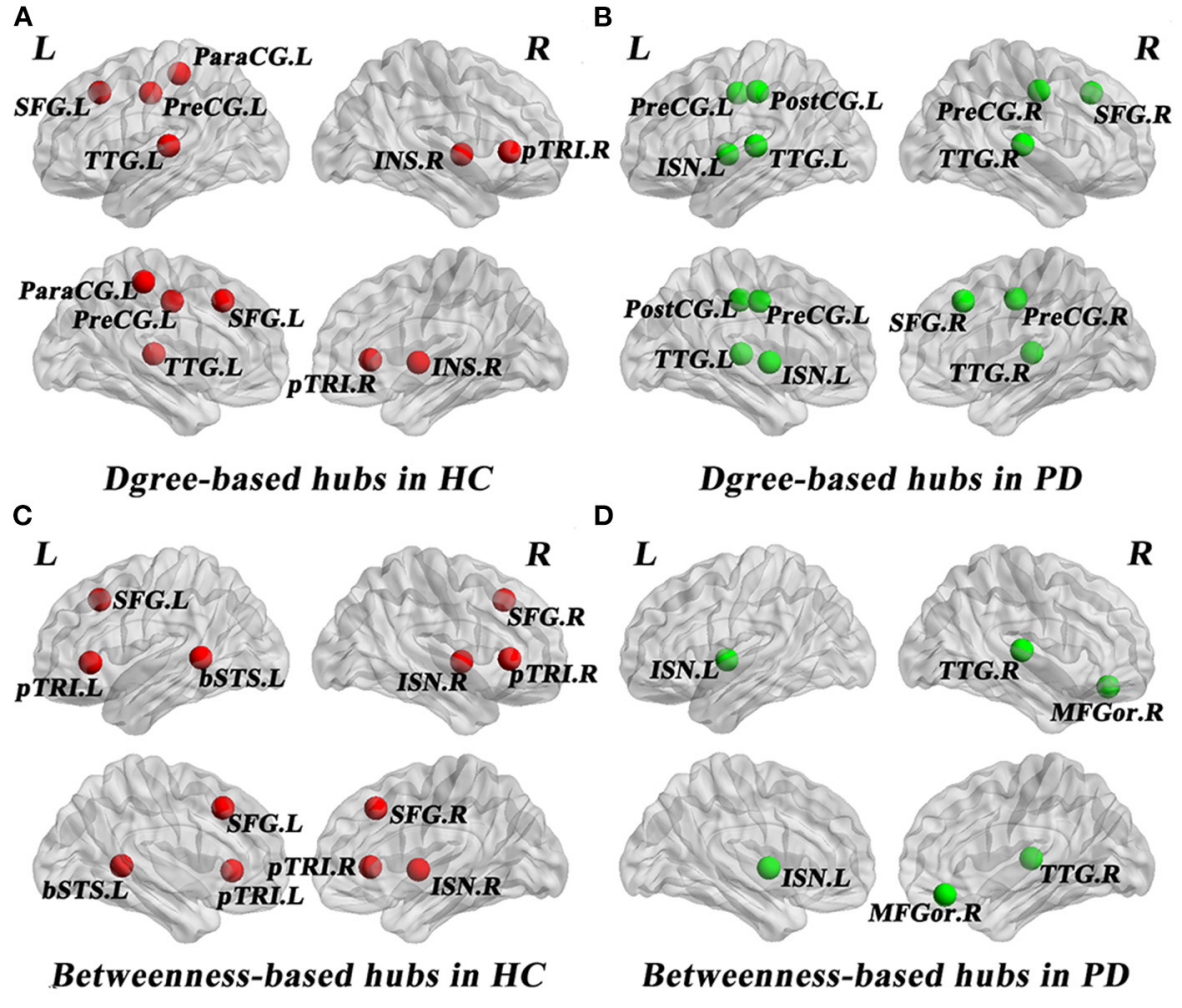
Network hubs in healthy controls (HC) and PD patients. Six degree-based hubs in HC **(A)**, 7 degree-based hubs in PD patients **(B)**, 6 betweenness- based hubs in HC **(C)**, and 3 betweenness-based hubs in PD patients **(D)**. All abbreviations were listed in Table 2.

As shown in Table 3, distinct distribution and number of degree-based and betweenness-based hubs were identified between the two groups. Based on nodal degree, six hub regions were identified in HC and seven hub regions were identified in PD patients. Based on nodal betweenness, six regions were identified as hubs in HC and three regions were identified as hubs in PD patients. These degree-based and betweenness-based hub regions of both groups were mainly located in frontal, temporal and insular lobes (Table 3, Figure 3).

**Table 3:** Hub distribution for each group.

**Hubs**	**HC**	**PD**
Degree-based hubs	L paracentral	L postcentral
	L precentral	L precentral
	L superiorfrontal	R precentral
	R parstriangularis	R superiorfrontal
	L transversetemporal	L transversetemporal
	R insula	R transversetemporal
		L insula
Betweenness-based hubs	L bankssts L parstriangularis	R medialorbitofrontalR transversetemporal
	R parstriangularis	L insula
	L superiorfrontal	
	R superiorfrontal	
	R insula	

*HC, healthy control; PD, Parkinson's disease; L, left; R, right*.

### Degree Distribution

**Figure 4:**
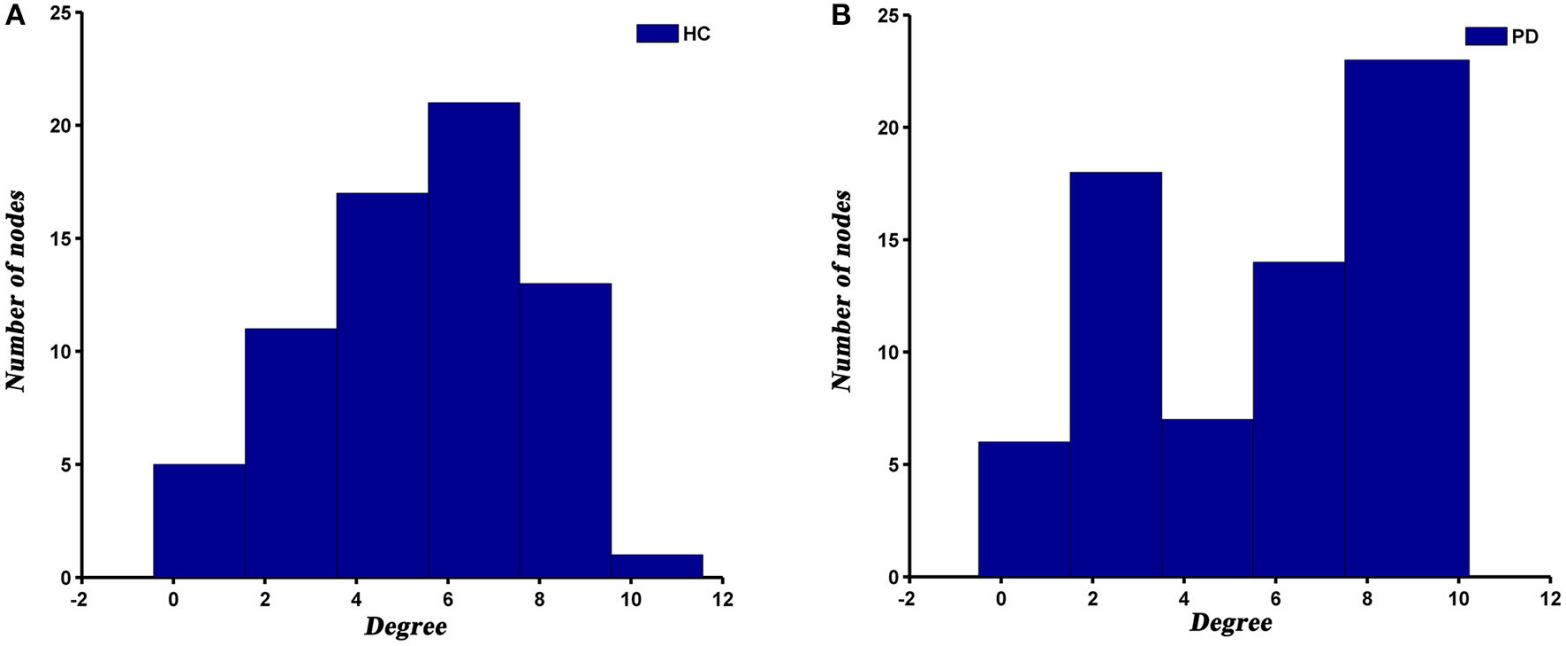
The histograms of degree distributions in HC **(A)** and PD **(B)** groups.

The degree distribution of both PD and HC networks followed an exponentially truncated power law. For the PD patients, the power exponent (k) was 1.04 and for HC was 1.28. The cut-off degree (dc) was 7.39 for PD and 4.75 for HC. The goodness of fit was 0.88 for PD patients and 0.82 for HC. The histograms of degree distributions showed more high-degree regions in PD patients compared to HC (Figure 4).

### Network Robustness

**Figure 5:**
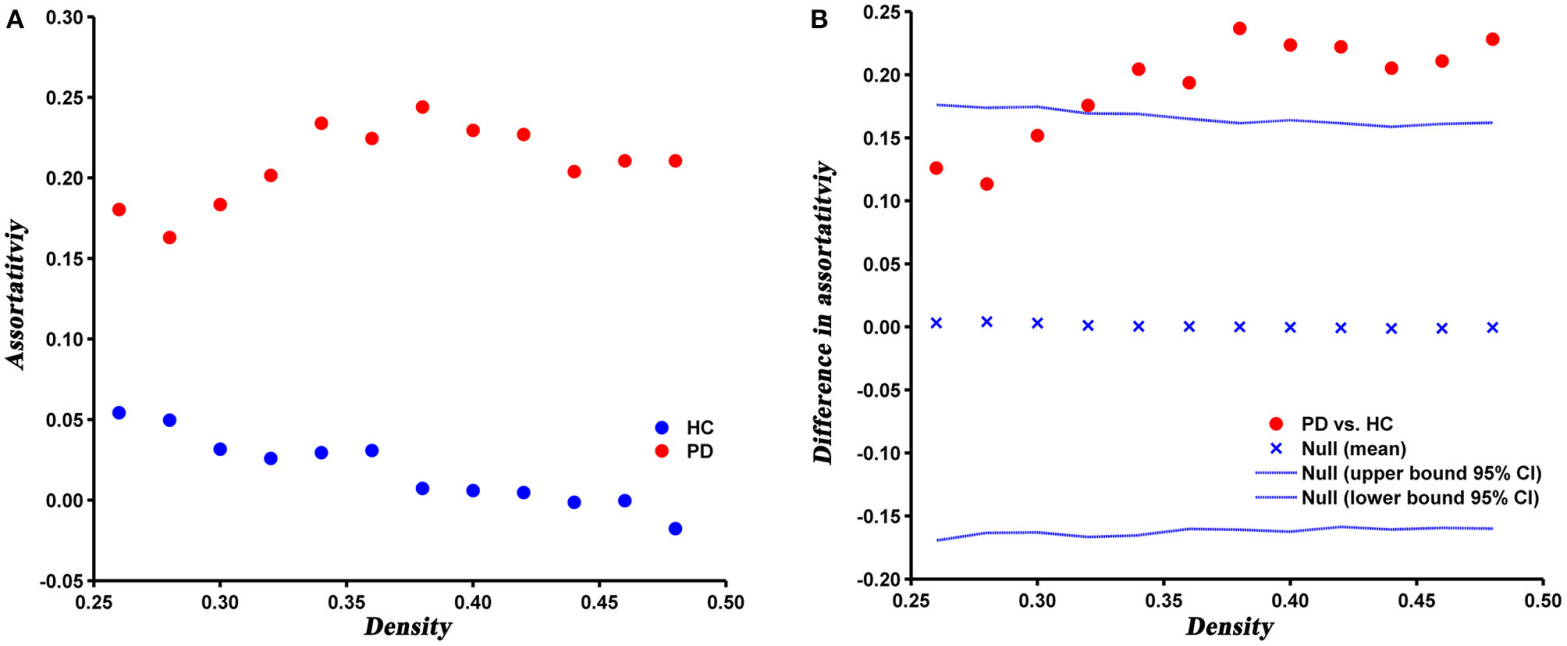
Changes **(A)** and between-group differences **(B)** of assortativity as a function of network density in HC and PD groups. Between-group differences (Dot markers) lying outside the 95% confidence intervals (dashed lines) indicate the densities where the difference is significant at *P* < 0.05. Positive values indicate densities where values for PD patients are greater than for HC and negative values indicate the opposite.

Compared with HC, the assortativity of PD patients was significantly increased, which was confirmed by the AUC analysis (*P* = 0.005) (Figure 5).

**Figure 6:**
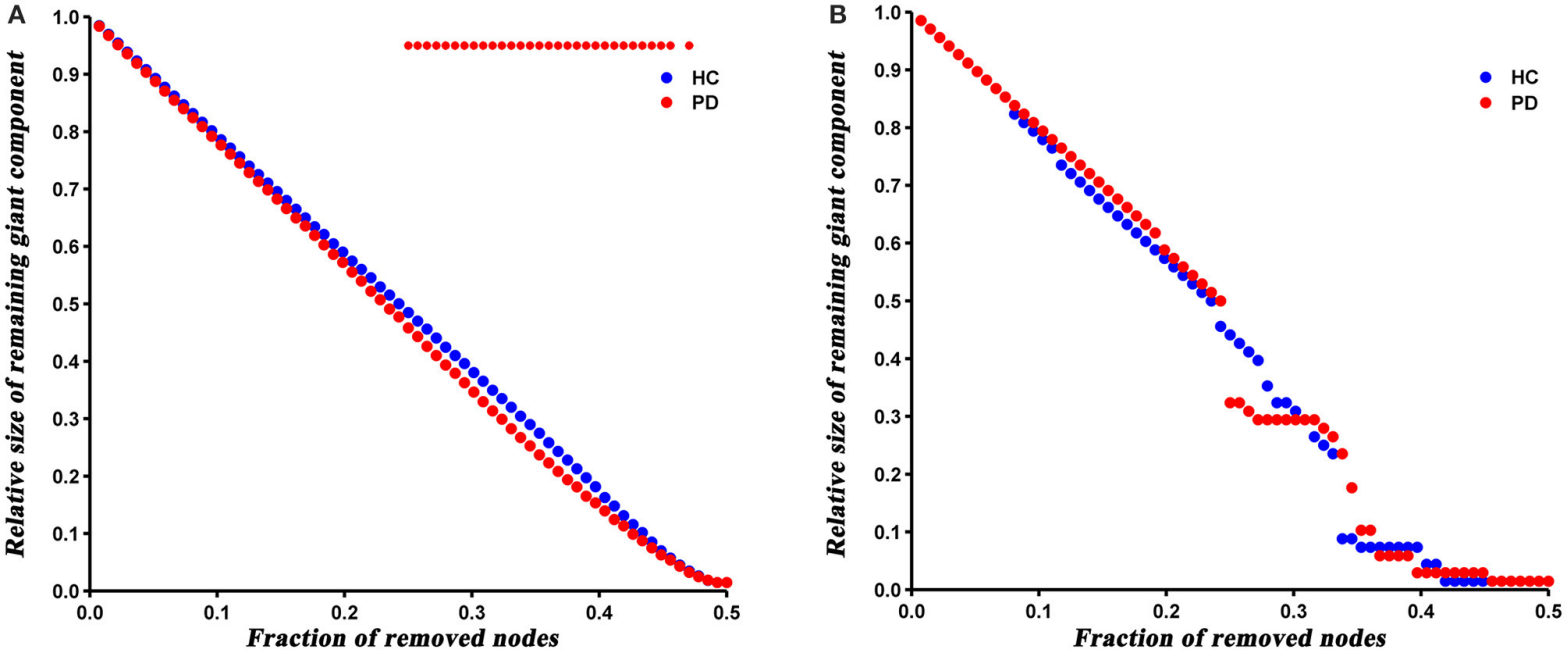
Results of network resilience to random failure **(A)** and targeted attack **(B)**. Red stars represent significant differences between HC and PD patients.

The robustness analysis revealed that PD patients were more vulnerable to a random failure (*P* = 0.023), but not a targeted attack (*P* = 0.421), compared to HC (Figure 6).

## Discussion

As far as we have known, it is the first study demonstrating abnormal topological organization of SD-based SCNs in PD patients compared to HC. Specifically, PD patients showed increased normalized clustering coefficient and normalized path length in global network parameters, as well as a reorganization of degree-based and betweenness-based hubs (i.e., less frontal hubs). Moreover, the degree distribution analysis showed more high-degree nodes in PD patients. In addition, we also found the increased assortativity and reduced robustness under a random attack in PD patients compared to HC. Taken together, these findings indicated an abnormal topological organization of SD-based SCNs in PD patients, which may contribute in understanding the pathophysiology of PD at the network level.

### Alterations in Global Network Parameters

The SCNs of both PD and HC groups have small-world topological properties, that is, higher normalized clustering coefficients and similar normalized path lengths than random networks. Small-world topological networks support local specialization and integration processing with efficient information transmission while minimizing wiring costs (Achard et al., 2006; Kaiser and Hilgetag, 2006). This finding is consistent with previous SCNs studies in PD patients (Pereira et al., 2015; Zhang et al., 2015; Xu et al., 2017, 2018; Guo et al., 2018; Wu et al., 2018). There is now strong evidence that the small-world property is usually a normal configuration of human brain networks.

However, compared with HC, PD patients showed significantly increased normalized clustering efficient and normalized path length, which indicated that local specialization is significantly enhanced and global information integration ability is reduced in PD patients. PD network tends to a more regularized network. Such abnormal topological organization may be due to the reduced long-distance connections and the increased short-distance connections as the local specialization and global information integration are mainly associated with short- and long-range connections, respectively (Latora and Marchiori, 2001; He et al., 2009). Our results are consistent with most of previous SCNs studies based on other structural features in PD. For example, in structural networks built with LGI, Xu et al. (2017) also found higher clustering coefficient and characteristic path length in PD patients compared with HC. However, some SCNs studies found no changes of clustering coefficient and characteristic path length in PD (Guo et al., 2018; Xu et al., 2018). Additionally, our findings are also in agreement with some DTI studies with regard to lower global efficiency(the inverse of path length) in PD patients compared to HC, but are inconsistent with the lower clustering coefficient (Kamagata et al., 2018; Vriend et al., 2018; Koirala et al., 2019; Hu et al., 2020).

These discrepancies among these results may be related to the heterogeneity of PD patients (i.e., different stages, disease duration or clinical phenotypes) and the variations in methodological approaches (e.g., different atlases and morphometric features). For instance, the early-stage PD patients were recruited in some studies and the pathological damage to brain structures may be not obvious, thus the SCNs can still maintain the normal information transfer in these studies (Guo et al., 2018; Xu et al., 2018). Moreover, cognitive impairment may be related to more severe topological network changes than normal cognition in PD patients. For instance, in structural networks built with cortical thickness and subcortical volume, Pereira et al. (2015) have found that PD patients with mild cognitive impairment show larger characteristic path length and reduced global efficiency compared with controls, while no significant differences of global network parameters were found between PD patients with normal cognition and controls. Similar findings were also observed in a white matter connectivity network study by Wang et al. (2019). In addition, different brain parcellation schemes (e.g., 68, 90, 116, 162, and 264 brain atlases) and different morphometric features (e.g., local gyrification index, gray matter volume, deformation-based morphometry, and cortical thickness) in previous studies may also contribute in the inconsistent results (Pereira et al., 2015; Zhang et al., 2015; Xu et al., 2017, 2018; Guo et al., 2018; Wu et al., 2018). However, the reasons for these discrepancies are not entirely clear and still need to be further investigated.

### Network Hubs

In the present study, distinct distribution and number of degree-based and betweenness-based hubs were identified between the two groups, indicating hub reorganization in PD patients. In particular, compared with HC, PD patients showed lost degree-based hubs in the left superiorfrontal, the right parstriangularis and betweenness-based hubs in the bilateral parstriangularis and the bilateral superiorfrontal, which may be related to the fronto-striatal dopaminergic deficits in PD patients (Seidler et al., 2010). In addition, the right transversetemporal was identified as a new hub in PD patients. These findings are consistent with a brain metabolic connectome using [18F] FDG-PET in early idiopathic PD patients, which found widespread long-distance connectivity decreases and loss of hub regions mainly in frontal cortex, as well as long-distance connectivity increases in parietal, occipital and temporal cortices (Sala et al., 2017). Another study of gray matter volume-based SCNs observed lost hubs mainly located in frontal cortex in PD (Guo et al., 2018), which was also in line with our results. As the frontal cortex is primarily responsible for cognitive function, the significant loss of hub regions in the frontal cortex may indicate cognitive impairment in PD patients. In addition, more degree-based hubs indicate more high-degree regions in PD patients compared to HC. However, less betweenness-based hubs in PD patients may reflect the increased separation as betweenness-based hubs play an important role in bridging nodes between different modules and facilitate the information transmission and integration of the whole network (Rubinov and Sporns, 2010).

### Degree Distribution

Previous studies have demonstrated that human brain networks followed an exponentially truncated power law distribution (He et al., 2007; Hosseini et al., 2016; Cao et al., 2019; Palaniyappan et al., 2019), which were consistent with our results. This degree distribution pattern showed that PD and HC networks were comprised of many nodes with relatively few connections and some hub nodes with many connections. Such a network configuration may be more resilient to a targeted attack than a scale-free network, which is characterized by the coexistence of some super hubs (i.e., nodes with an extremely high degree or betweenness) and a large number of non-hub nodes (Albert et al., 2000; Achard et al., 2006). However, although the degree distribution patterns of both groups were basically similar, there were also visible differences between them. We observed a higher cut-off of the degree in PD patients relative to HC, suggesting more high-degree nodes in PD patients. This subtle change may indicate that the SCNs of PD is less tolerant to a targeted attack than HC.

### Network Robustness

Assortativity can directly reflects the robustness of the network. In the present study, the assortativity was significantly increased in PD patients relative to HC, which suggested nodes with the same degree were more closely interconnected, that is, high-degree nodes were connected to other nodes with high-degree and low-degree nodes were connected to other nodes with low-degree. It has been suggested that network with moderately increased assortativity are more robust to a targeted attack, but relatively less robust to a random attack (Trajanovski et al., 2013). In a higher assortative network, high-degree nodes are closely connected with each other, thus forming a centralized interconnection core. When a node in the core is removed, the other nodes in the core can still maintain maximum connectivity of the network. Therefore, the network is relatively robust against a targeted attack. However, as the majority of low-degree nodes tend to connect to other low-degree nodes, the network is more prone to fragmentation under a random attack (Iyer et al., 2013). In addition, we speculated that the higher assortativity of PD network may be mainly due to the increased clustering coefficient, since the clustering coefficient has a positive contribution to the assortativity of a network (Estrada, 2011).

At last, we analyzed the network robustness of both groups under direct random or targeted attacks. The results showed that PD patients had similar robustness to that of HC network in the case of target attack, but significantly reduced robustness under a random attack. It has been shown that the robustness of a network under random or targeted attacks depends greatly on its topological properties (Iyer et al., 2013). As described above, the degree distribution analysis showed more high-degree nodes in PD patients, indicating that PD network is less tolerant to a targeted attack than HC. Nevertheless, these high-degree nodes were closely interconnected (increased assortativity), which resulted in enhanced robustness of the PD network under a targeted attack. Hence, we speculated that the offsetting effect of both topological properties may result in the similar target attack tolerance of both groups. Similarly, the number of low-degree nodes in PD network increased and connected to each other, so the network was more likely to fragment in the event of a random attack.

## Limitations

This study has several limitations that need to be addressed. First, the cross-section design of the study does not allow the characterization of the dynamic changes of SD-based SCNs in PD patients. Second, the network parameters were calculated at the group level, so the relationship between clinical indices and network parameters cannot be discussed. Finally, most of the PD patients in our study had a long-term history of dopaminergic medications, which may lead to structural plasticity and the corresponding SCNs changes. Future studies of early drug-naive PD patients are warranted.

## Conclusion

In this study, we have applied SD and graph theoretical analysis to investigate the topological alterations of SCNs in PD patients. We revealed an abnormal topological organization in PD patients as evidenced by increased normalized clustering coefficient and normalized path length, a reorganization of hub and degree distribution, increased assortativity and reduced robustness under a random attack in PD patients compared to HC. These findings may contribute in understanding the pathophysiology of PD at the network level.

## Data Availability Statement

The raw data supporting the conclusions of this article will be made available by the authors, without undue reservation.

## Ethics Statement

The studies involving human participants were reviewed and approved by the Medical Research Ethical Committee of The Second Affiliated Hospital of Soochow University. The participants provided their written informed consent to participate in the study.

## Author Contributions

GF and ZJ designed and organized the study. YY, JX, CM, and WL collected and analyzed the data. EW and YJ drafted and revised the manuscript. All authors approved the submitted version.

## Conflict of Interest

The authors declare that the research was conducted in the absence of any commercial or financial relationships that could be construed as a potential conflict of interest.
